# Specific Imaging of Inflammation with the 18kDa Translocator Protein Ligand DPA-714 in Animal Models of Epilepsy and Stroke

**DOI:** 10.1371/journal.pone.0069529

**Published:** 2013-08-02

**Authors:** Denise Harhausen, Violetta Sudmann, Uldus Khojasteh, Jochen Müller, Marietta Zille, Keith Graham, Andrea Thiele, Thomas Dyrks, Ulrich Dirnagl, Andreas Wunder

**Affiliations:** 1 Department of Experimental Neurology, Charité – Universitätsmedizin Berlin, Berlin, Germany; 2 Center for Stroke Research Berlin (CSB), Berlin, Germany; 3 Global Drug Discovery, Bayer Healthcare, Berlin, Germany; Julius-Maximilians-Universität Würzburg, Germany

## Abstract

Inflammation is a pathophysiological hallmark of many diseases of the brain. Specific imaging of cells and molecules that contribute to cerebral inflammation is therefore highly desirable, both for research and in clinical application. The 18 kDa translocator protein (TSPO) has been established as a suitable target for the detection of activated microglia/macrophages. A number of novel TSPO ligands have been developed recently. Here, we evaluated the high affinity TSPO ligand DPA-714 as a marker of brain inflammation in two independent animal models. For the first time, the specificity of radiolabeled DPA-714 for activated microglia/macrophages was studied in a rat model of epilepsy (induced using Kainic acid) and in a mouse model of stroke (transient middle cerebral artery occlusion, tMCAO) using high-resolution autoradiography and immunohistochemistry. Additionally, cold-compound blocking experiments were performed and changes in blood-brain barrier (BBB) permeability were determined. Target-to-background ratios of 2 and 3 were achieved in lesioned vs. unaffected brain tissue in the epilepsy and tMCAO models, respectively. In both models, ligand uptake into the lesion corresponded well with the extent of Ox42- or Iba1-immunoreactive activated microglia/macrophages. In the epilepsy model, ligand uptake was almost completely blocked by pre-injection of DPA-714 and FEDAA1106, another high-affinity TSPO ligand. Ligand uptake was independent of the degree of BBB opening and lesion size in the stroke model. We provide further strong evidence that DPA-714 is a specific ligand to image activated microglia/macrophages in experimental models of brain inflammation.

## Introduction

In diseases primarily characterized by neuroinflammation, such as multiple sclerosis, encephalitis, or meningitis, infiltration of immune cells into the brain is accompanied by breakdown of the blood-brain barrier (BBB) and substantial glial activation triggered by an autoimmune reaction and microbial infection, respectively [1], [2]. Brain inflammation and BBB breakdown also play a significant role in stroke [3], epilepsy [4], and traumatic brain injury [5]. Methods that allow the noninvasive, specific monitoring of brain inflammation in animal models and in patients are of high value.

A number of approaches to imaging inflammation and related pathology in the brain have been developed for use in animal models as well as in patients. They range from magnetic resonance imaging (MRI) to nuclear imaging techniques such as positron emission tomography (PET) and single-photon emission computed tomography (SPECT). For example, BBB impairment as a part of the inflammatory process can be visualized by systemic injection of imaging agents that, under physiological conditions, do not cross the intact BBB (e.g. gadolinium-based contrast agents for MRI or radiolabeled small molecules for PET or SPECT). Another approach is to target leukocytes of the blood stream, either by systemic application or re-injection after extracorporal labeling with paramagnetic or radioactive agents. Subsequently, the homing of these cells towards the brain lesion can be visualized [6], [7], [8].

One target used frequently for specific imaging of cerebral inflammation is the 18 kDa translocator protein (TSPO), formerly known as peripheral benzodiazepine receptor (PBR). Under physiological conditions, TSPO expression in the brain is relatively low and can be detected mostly in ependymal cells lining the ventricles, in cells of the olfactory bulb and the choroid plexus as well as in resting glial cells, including microglia and astrocytes. In response to cerebral inflammation triggered by brain injury, TSPO expression increases markedly in activated glial cells, especially microglia, and in blood-borne macrophages/monocytes infiltrating the lesion [9]. Numerous radiolabeled TSPO ligands have been developed and evaluated in animal models of disease and in humans; of these [^11^C]PK11195 is the ligand most extensively studied [10], [11]. Several other ligands with improved properties (e.g. higher affinity, lower lipophilicity) have been described including [^11^C]vinpocetin [12], [^18^F]FMDAA1106, [^18^F]FEDAA1106 [13], [^123^I]CLINDE [14], [^11^C]CLINME [15], [^18^F]FEPPA or [^18^F]PBR28 [16], [^11^C]DAC [17], and [^11^C]DAA1106 [18]. The pyrazolopyrimidine [^18^F]DPA-714 was introduced in 2008 by James and colleagues as a highly specific new radioligand for TSPO with improved imaging properties over [^11^C]PK11195 [19]. [^18^F]DPA-714 has been successfully evaluated for the specific imaging of inflammation in various models of neuroinflammation [18], [20], [21], [22], [23] and in a brain tumor model [24]. In healthy humans, the compound showed excellent *in-vivo* stability and biodistribution as well as an acceptable effective dose estimation [25]. These studies raise hope that [^18^F]DPA-714 might be used as a marker to specifically detect CNS inflammation in humans.

In contrast to the given publications [22], [23], the aim of this study was to evaluate the specificity of [^18^F]DPA-714/ [^3^H]-DPA-714 by (i) correlating high-resolution autoradiography with immunohistochemistry and (ii) addressing the impact of blood-brain barrier changes. Therefore, animal models of CNS injury with different neuroinflammatory responses were used and [^18^F]DPA-714/ [^3^H]-DPA-714 signal was correlated to different outcome measurements, which can influence tracer binding. Secondary chronic CNS inflammation was investigated in a rat epilepsy model generated by systemic injection of the excitotoxin Kainic acid (KA) [26]. The acute inflammatory response was modeled using transient middle cerebral artery occlusion (tMCAO) in the mouse, a model of ischemic stroke that is characterized by infarction and glia cell activation as well as invasion of activated blood-borne cells. The specificity of the tracer binding towards activated microglia/macrophages was determined at the cellular level by high-resolution microautoradiography. In addition, BBB disturbances and lesion volume are both parameters that could influence unspecific tracer extravasation and uptake. Therefore, those parameters were evaluated in the stroke model where complex changes of the BBB occur and subside within days as the lesion evolves.

## Materials and Methods

### Animals and ethical statement

All procedures were performed in accordance with the German animal welfare laws (Landesamt fuer Gesundheit und Soziales Berlin) and approved under the animal research licenses A0189/09 and A0920, respectively. Local guidelines were adhered to with respect to premature euthanasia when animals showed signs of distressed according to our daily heath check. Animals were housed under standard conditions with free access to food and water.

### Radiochemical synthesis

The radioligand [^18^F]DPA-714 was synthesized as previously described [19]. [^3^H]DPA-714 was synthesized from a brominated derivative that was generated *in situ* from DPA-714 (synthesized according to James and colleagues [19]) using bromine (1 equiv) in glacial acetic acid for 30 min at room temperature (LC-MS showed the desired brominated product). The reaction was concentrated and dissolved in acetonitrile to generate a 10 mg/ml stock solution. Tritiation was performed according to the literature [27] using tritium gas and palladium(II) hydroxide with triethylamine in acetonitrile after HPLC purification [^3^H]DPA-714 with a specific activity of 880 GBq/mmol (23.8 Ci/mmol). ^3^H-NMR spectroscopy showed that 50% of the tritium was N,N-diethyl-2-{2-[4-(2-fluoroethoxy)phenyl]-5,7-dimethyl(3H)pyrazolo[1,5-a]pyrimidin-3-yl}acetamide and approximately 11% N,N-diethyl-2-{2-[4-(2-fluoroethoxy)(2-3H)phenyl]-5,7-dimethylpyrazolo[1,5-a]pyrimidin-3-yl}acetamide.

### Kainic acid model in rats, administration of the compounds, and autoradiography

The Kainic acid model in male Spraque-Dawleys rats was performed as previously described [28]. In order to induce epileptic seizures, secondary inflammation, and microglia activation, rats (n = 5) received 9 mg/kg Kainic acid intraperitoneally on day 0, and 4.5 mg/kg Kainic acid on days 3 and 7. To evaluate the lesion and to exclude healthy rats, the animals were examined with T1- and T2-weighted MRI at days 1 and 7 after Kainic acid treatment.

At days 8 and 10, rats received [^18^F]DPA-714 alone (n = 2) or were co-injected with the TSPO ligands FEDAA1106 (1.6 mg/kg; n = 1) and DPA-714 (0.95 mg/kg, n = 2), respectively (100-fold excess). Agents were administered intravenously at a dose of 25 to 35 MBq. Thirty minutes after tracer injection, rats were euthanized by decapitation, brains were removed, snap frozen in −40°C isopentane, sliced using a cryostat (Leica, CM3000), and exposed overnight to PhosphorImager plates (BAS-IP SR 2025, FUJI, Duesseldorf, Germany). After exposure, the sections were fixed with paraformaldehyde and immunohistochemically stained with an anti-Ox42 antibody (MCA711, Serotec, Duesseldorf, Germany), as described below. In order to determine target-to-background ratios (TBRs) of [^18^F]DPA-714 uptake in the hippocampus, region-of-interest analysis (ROI) was performed using ImageJ (version 1.45) with the cerebellum as a reference region.

### Focal cerebral ischemia in mice, administration of the compounds, and autoradiography

Transient middle cerebral artery occlusion (tMCAO) was induced in adult male C57BL6/N mice (Charles River, Sulzfeld, Germany) at the age of 10 weeks by inserting a silicone-coated 8/0 nylon monofilament (Xantopren M Mucosa and Activator NF Optosil Xantopren, Heraeus Kulzer, Wehrheim, Germany) via the internal carotid artery as described by Engel and colleagues [29]. In sham controls, surgery was performed, the filament inserted but immediately retracted. Mice were anesthetized with isoflurane (2% for induction and 1.5% for maintenance) under 70% N_2_O and 30% O_2_ via a face mask. Duration of anesthesia did not exceed 10 min. After 30 min of ischemia, animals were re-anesthetized and the filament removed to permit reperfusion. Mice were treated with Lidocain (Xylocain) as a local anesthesia directly after and around 6 h after surgery. During surgery and ischemia, body temperature was measured and kept constant between 37.0 and 37.5°C with a heating pad. After surgery, the animals were allowed to wake up in a warming cage and kept there for around 2 h.

Mice were injected intravenously with 2 MBq of [^3^H]DPA-714 at 6 h (n = 5), 24 h (n = 5), 96 h (n = 28), or 2 weeks (n = 5) after reperfusion. To determine the best time point of tracer injection (best tracer uptake time), mice with 96 h of reperfusion were sacrificed 30, 60, or 90 min after injection of [^3^H]DPA-714. Since 60 min turned out to be the best time point, all the other groups (6 h, 24 h, and 2 weeks) were scarified at this time point after tracer injection. To block tracer binding, a subgroup of animals (n = 10; 96 h after reperfusion) was pre-injected with a 100-fold excess of PK11195 or DPA-714 one hour before injection of [^3^H]DPA-714. Mice were sacrificed by decapitation at 60 min after tracer injection with an additional injection of 2 mg/kg FITC-labeled bovine serum albumin (FITC-BSA, Molecular Probes, Darmstadt, Germany) 30 min before sacrifice. Brains were removed, snap frozen in ice-cold methyl butane, and coronal cryosections with a thickness of 20 µm were cut at interaural positions 6.6, 5.3, 3.9, 1.9, and 0 mm (several series of each brain). One series of slices was incubated for 4 weeks on a tritium-sensitive imaging plate (Fuji Imaging plate; BAS-IP TR 2025, Duesseldorf, Germany) and digitalized with Bio-Imaging Analyzer BAS 5000 (Fuji, Duesseldorf, Germany). TBRs were calculated by ROI analysis (ipsilateral areas of the damaged striatum and cortex versus mirrored contralateral areas) using ImageJ. After autoradiography, the slices were stored at −20°C.

### BBB impairment and infarct volumetry in the experimental stroke model

BBB impairment and infarct volumetry was evaluated using the same series of brain slices that were used for autoradiography. BBB impairment was analyzed by detection of FITC-BSA in the cortex and striatum using a fluorescence microscope (Zeiss Axio Imager Z1, Jena, Germany). The FITC-BSA signal in the lesion area was evaluated by two independent investigators and classified as low, medium, or high intensity. These qualitative estimates were then correlated to the TBRs. On the same sections, infarct volumetry was performed, and adjacent sections were used to analyze the number of activated microglia using Iba1 staining (see below). Infarct volumes were determined with hemalaun staining. After fluorescence microscopy, sections were rinsed in an alcohol series of decreasing concentrations before incubation with hemalaun (Merck, Darmstadt, Germany) for 5 min. The slides were then rinsed in 96% ethanol with 2% hydrochloric acid and incubated in 2% sodium bicarbonate. After dehydration and mounting, the sections were analyzed with a LEICA DMRE microscope. The sections were digitized and the area of infarction (as defined by a lower overall cell density because of cell damage) quantified using Sigma Scan Pro, Version 5.0.0 (Jandel Scientific, San Rafael, CA, USA). A correction for edema was applied by calculating the ‘indirect’ infarct volume as the volume of the contralateral hemisphere minus the non-infarcted volume of the ipsilateral hemisphere.

### Immunohistochemical staining of Iba1 and Ox42

In tMCAO animals, immunohistochemical staining was performed on sections adjacent to the ones used for autoradiography and in Kainic acid-treated animals, the same sections used for autoradiography were used for immunohistochemistry. Sections were fixed in 4% PFA, and endogenous peroxidase activity was blocked by incubating with peroxidase block (Dako, Hamburg, Germany) for 20 min. The sections were then incubated in blocking solution containing 3% normal donkey serum (Jackson ImmunoResearch, West Grove, PA, USA) and 0.3% Triton X-100 (Sigma-Aldrich, Seelze, Germany) in PBS for 60 min. The primary antibody rabbit anti-Iba1 (Wako-chemicals, Neuss, Germany; 1∶200) was applied for 3 h at room temperature. The tissue was then rinsed three times with PBS for 5 min. The secondary antibody donkey anti-rabbit IgG biotin (Jackson ImmunoResearch, West Grove, PA, USA; 1∶500) was applied to the sections for 60 min. After 3 washings, the ABC kit (Vector, Burlingame, USA) was applied according to the manufacturer instructions followed by the substrate kit VIP (Vector, Burlingame, USA). Sections were mounted and analyzed with a LEICA DMRE microscope. The same procedure was used to stain rat sections with Ox42 antibody. The primary antibody anti-Ox42 (MCA711 Serotec, Duesseldorf, Germany, 1∶100) was incubated overnight at 4°C. The sections were washed and incubated with the secondary antibody donkey-anti rat IgG biotin (Jackson ImmunoResearch, West Grove, PA, USA; 1∶500) for 1 h, and developed with the ABC Kit and VIP Kit as described above.

### Microautoradiography in the stroke model

For high resolution microautoradiography, brain sections adjacent to the Iba1-stained sections were used. This was necessary, since the immunohistochemical procedure led to a signal decrease on the autoradiography section and vice versa the fixation necessary for immunohistochemistry disturbed ligand binding. For high-resolution microautoradiography, the sections were first incubated for 5 min in 25 mM HEPES Buffer, then incubated with 100 Bq/µl [^3^H]DPA-714 (diluted in 25 mM HEPES Buffer and 0.1% BSA) for one hour at room temperature. The sections were then washed three times in 25 mM HEPES Buffer and 0.1% BSA and rinsed once in water. After drying for around 1 h, they were protected from light, dipped in NTB-Photoemulsion (Kodak, Stuttgart, Germany), dried, and exposed for 4 weeks while protected from light at 4°C. The slides were developed in Phenisol solution (ILFORD, Marly, Switzerland) and fixed in HYPAM solution (ILFORD, Marly, Switzerland). Sections were mounted with the aqueous mounting medium Aquatex® and analyzed using Axioplan Imager Z1 (Zeiss, Jena, Germany) microscope.

### Statistics

Normality was tested using Kolmogorov-Smirnov test and variance homogeneity using Levené test. When data were normally distributed and variance homogeneity was met, TBRs of [^3^H]DPA-714 were compared using two-way analysis of variance (ANOVA), followed by a post hoc Bonferroni test (using SPSS v.19.0). In case of violation of normality or variance homogeneity, Kruskal-Wallis test was performed. Correlations between TBRs and infarct volumes or FITC-BSA signal, respectively, were analyzed using linear regression models. Data are expressed as means ± SD and differences are considered significant at p<.05.

## Results

### [^18^F]DPA-714 in the rat Kainic acid model

**Figure 1:**
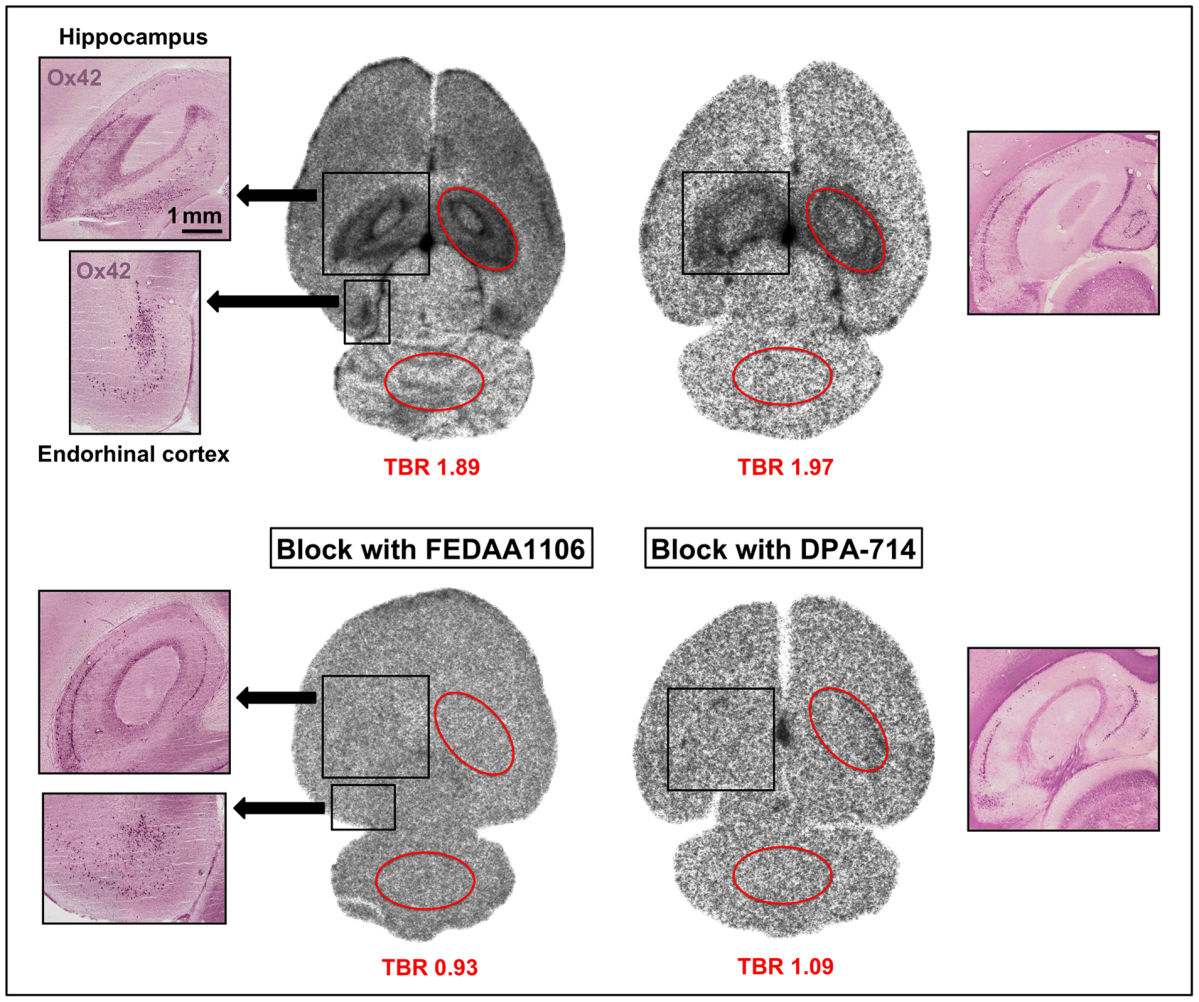
Distribution of [^18^F]DPA-714 in the rat Kainic acid model. [^18^F]DPA-714 was i.v. injected in rats 8 and 10 days after treatment with Kainic acid (epilepsy model) and its distribution in the brain and the effect of pretreatment with FEDAA and DPA-714 were analyzed. High-resolution autoradiographs of tracer distribution (black-and-white images) and anti-Ox42 stained sections of the same sections (color) are illustrated. The images in the upper row show that areas with high tracer binding (hippocampus and Entorhinal cortex) match anti-Ox42 staining for activated microglia/macrophages. TBRs were 1.89 and 1.97, respectively, ROIs are outlined in red. In the lower row, images of animals pretreated with FEDAA1106 and DPA-714 with positive staining for anti-Ox42 are depicted. Tracer binding is almost completely inhibited by both compounds (TBRs equal about 1).

[^18^F]DPA-714 was found to be enriched in all regions known to be affected by Kainic acid treatment: the hippocampal area as well as in cingulated and Entorhinal cortices (Fig. 1 upper row). The binding pattern corresponded well with anti-Ox42 immunoreactivity confirming the presence of activated microglia/macrophages. Accumulation of [^18^F]DPA-714 was completely blocked by co-injection with an excess of unlabeled FEDAA1106 (Fig. 1, lower row left) or unlabeled DPA-714 (Fig. 1, lower row right), respectively.

### [^3^H]DPA-714 in the mouse model of stroke

**Figure 2:**
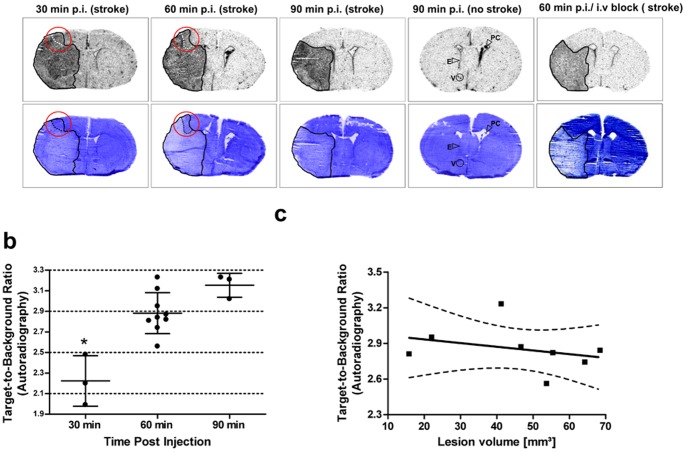
Distribution of [^3^H]DPA-714 96 h after tMCAO in mice. [^3^H]DPA-714 was i.v. injected in mice with transient middle cerebral artery occlusion (tMCAO) at 96 h after stroke and its distribution in the brain was analyzed. Autoradiographs (upper row) and hemalaun stained sections (lower row) at 30, 60, and 90 min after tracer injection and with pre-injection of PK11195 (right side) are depicted (**a**). Areas with increased tracer uptake matched well with the stroke area (outlined in black), except in some cases where increased tracer uptake was also detected in ipsilateral cortical regions right next to the stroke area (dotted lines in the red circle). Apart from lesioned tissue, increased tracer uptake was also seen in the plexus choroideus (PC), the ependyma (E), and in larger vessels (V) as indicated by the arrows (sham animal). In **b**, TBRs calculated from a ROI analysis (ipsilateral versus mirrored contralateral areas) are shown. The data was normally distributed (Kolmogorov-Smirnov test, Z = .67, p = .76) and variances were homogenous across groups (Levené test, F(2,12) = .40, p = .68). The overall TBR increased significantly from about mean ± SD of 2.2±.25 at 30 min to 2.9±.20 and 3.2±.16 at 60 and 90 min post injection, respectively (ANOVA, post hoc Bonferroni test, *p<.05). There was no significant difference between cortical and striatal areas (data not shown). In **c**, TBRs are shown to be unaffected by lesion volume (stroke mice 60 min post injection, linear regression: R^2^ = .10, F(1,6) = .63, p = .46).

Mice with tMCAO were sacrificed at 30, 60 or 90 min post [^3^H]DPA-714 injection, autoradiography was performed, TBRs were calculated for cortical and striatal areas, and the same sections were stained with hemalaun (Fig. 2). Increased tracer uptake was detected throughout the whole stroke area at all time points (Fig. 2a, upper row), exceeding the lesion in some cases (Fig. 2a dotted lines within the red circles). Increased [^3^H]DPA-714 uptake was also detected in structures with constitutive TSPO expression, namely in larger vessels (V), ependymal cells (E), and cells of the plexus choroideus (PC), which is most clearly visible in the sham animal (Fig. 2a, upper row, right). TBRs between the ipsi- and contralateral cortex and striatum at the three different time points after tracer injection were compared. The data was normally distributed (Kolmogorov-Smirnov test, Z = .67, p = .76) and variances were homogenous across groups (Levené test, F(2,12) = .40, p = .68). TBRs between striatum and cortex were not different (data not shown). The overall TBR significantly increased from about mean of 2.2±.25 at 30 min to 2.9±.20 and 3.2±.16 at 60 and 90 min post injection, respectively (Fig. 2b) (ANOVA, post hoc Bonferroni test, p<.05). There was no correlation of lesion size with TBRs (linear regression: R^2^ = .10, F(1,6) = .63, p = .46, see Fig. 2c).

**Figure 3:**
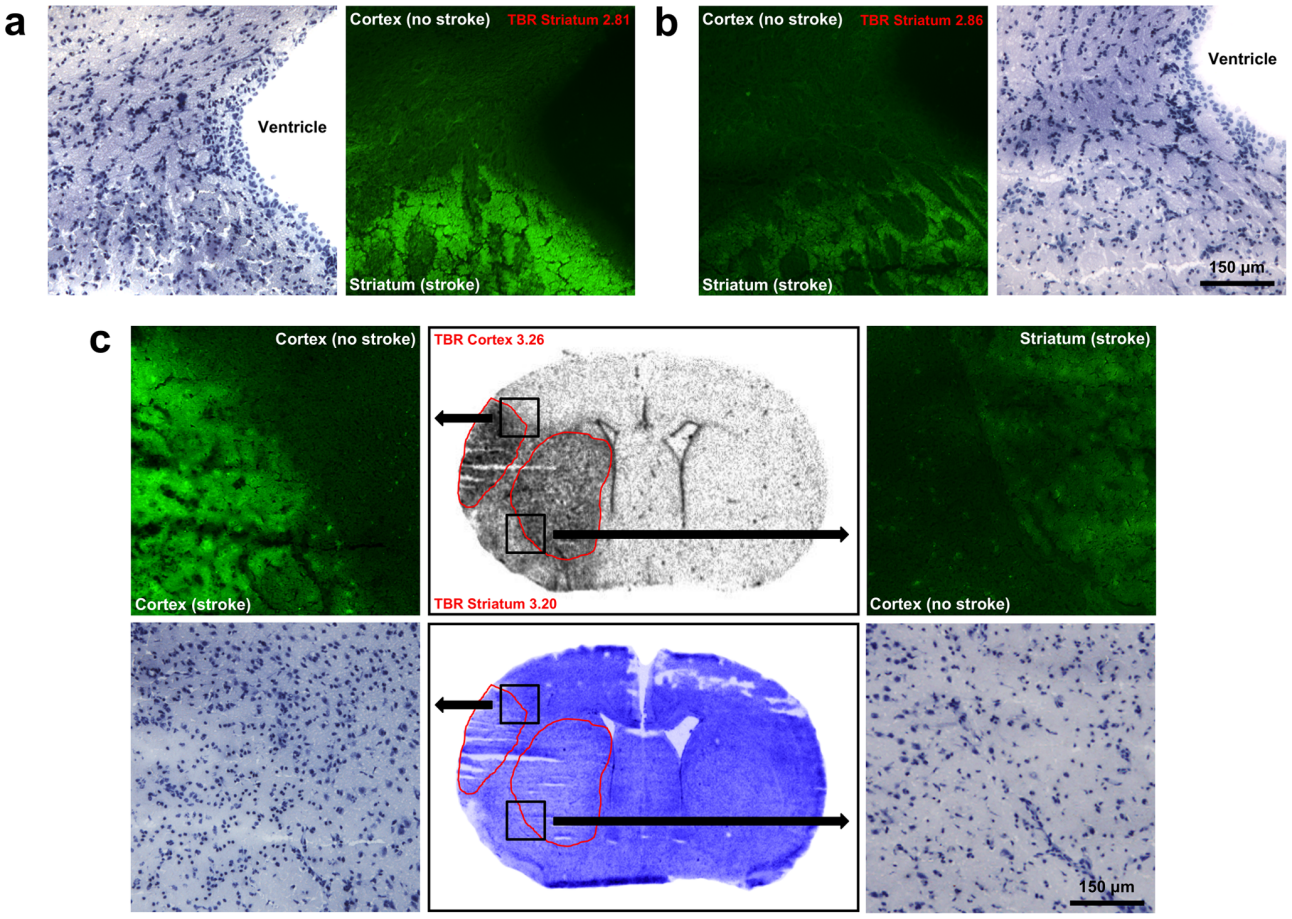
Comparison of BBB impairment and [^3^H]DPA-714 in mice with tMCAO. BBB impairment detected by fluorescence microscopy after i.v. injection of FITC-BSA was compared with [^3^H]DPA-714 in tMCAO mice. Fluorescence images of tissue sections (green color), autoradiography, and images of the same sections after hemalaun staining are depicted. In **a**, FITC-BSA extravasation is strong, whereas **b** shows an example of a mouse with a similar TBR, but weak FITC-BSA extravasation. **c** demonstrates a high resolution autoradiograph (middle, upper row) and the same section after hemalaun stain (middle, lower row) as well as fluorescence and hemalaun staining of two selected areas (black boxes). In this mouse, the ipsilateral cortex showed high fluorescence intensity, but only weak in the ipsilateral striatum. Similar TBRs were detected between the ipsilateral versus contralateral cortex/striatum. There was no correlation between FITC-BSA signal and TBRs (linear regression: R^2^ = .26, F(1,10) = 3.49, p = .09).

In order to investigate whether the [^3^H]DPA-714 signal is influenced by BBB impairment, FITC-BSA was injected 30 min prior to sacrifice. No correlation between BBB disturbance and TBRs was found (linear regression: R^2^ = .26, F(1,10) = 3.49, p = .09). Some animals with similar FITC-BSA fluorescence intensities showed different TBRs, while others showed comparable TBRs but different FITC-BSA intensities. The latter is depicted in Fig. 3 showing sections from three representative mice.

To assess the specificity of [^3^H]DPA-714 in the stroke model, a 100-fold excess of cold compound (DPA-714 or PK11195) was injected 1 h before application of [^3^H]DPA-714. The signal was clearly blocked. However, this did not affect the TBRs, since the intensities of [^3^H]DPA-714 signal decreased equally in both hemispheres (Fig. 2a).

**Figure 4:**
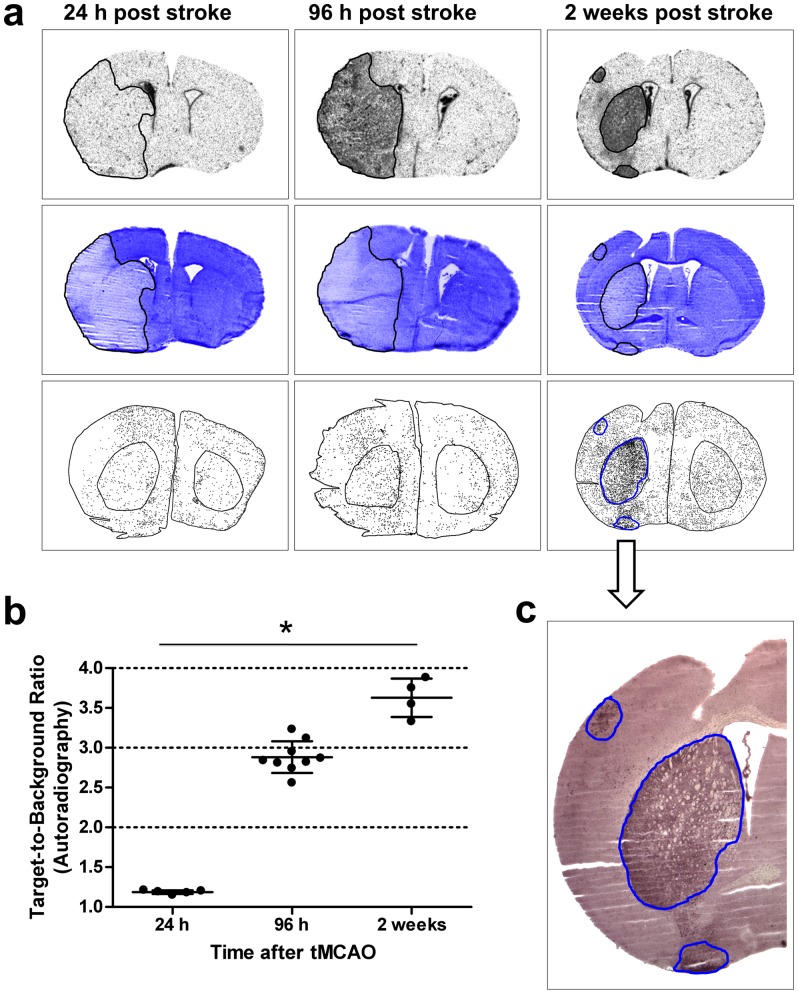
Uptake of [^3^H]DPA-714 in the brain of tMCAO mice at different time points after stroke. In **a**, high-resolution autoradiographs (upper row), the same sections after hemalaun staining (middle row), and the distribution of activated microglia/macrophages in the following sections as detected by anti-Iba1 staining (lower row) at 24 h, 96 h, and 2 weeks after stroke is shown. In **b**, TBRs calculated from a ROI analysis (ipsilateral versus mirrored contralateral areas) are depicted. Mean TBRs were found to be 1.19±0.02 at 24 h after stroke, 2.88±.20 at 96 h, and 3.63±.24 at 2 weeks. The data was normally distributed (Kolmogorov-Smirnov test, Z = 1.00, p = .27), but variances were not homogenous across groups (Levené test, F(2,15) = 3.60, p = .053). Mean TBRs were significantly different at all three time points (Kruskal-Wallis test, * p<.05). No differences were found between TBRs in cortical and striatal areas (data not shown). In **c**, an anti-Iba1 stained section of a mouse 2 weeks post stroke is shown. This demonstrates that areas with increased tracer binding perfectly match anti-Iba1 staining and the stroke area.

[^3^H]DPA-714 uptake was also investigated at different time points after tMCAO (Fig. 4). At early time points (6 h and 24 h after stroke), mean TBRs were 1.19±.02, indicating equal tracer uptake in lesioned tissue and normal tissue (data at 6 h not shown). This corresponded well with the absence of increased Iba1-immunoreactivity. At 96 h and two weeks after tMCAO, increased tracer uptake in the stroke area became clearly visible with mean TBRs of 2.88±.20 at 96 h and 3.63±.24 at 2 weeks. The data was normally distributed (Kolmogorov-Smirnov test, Z = 1.00, p = .27), but variances were not homogenous across groups (Levené test, F(2,15) = 3.60, p = .053). Mean TBRs were significantly different at all three time points (Kruskal-Wallis test, p<.05). Again, TBRs between striatum and cortex were not different (data not shown). Accordingly, larger numbers of Iba1-immunoreactive cells were present at these time points in the ischemic area, especially at 2 weeks. At 96 h and 2 weeks post ischemia, the areas of increased [^3^H]DPA-714 signal perfectly matched the region of Iba1-positive cells and the hemalaun staining. This pattern fits very well with known upregulation of TSPOs on activated microglia/macrophages. It should be noted that increased [^3^H]DPA-714 binding and Iba1 staining was found spreading to the perilesional rim.

### Microautoradiography in the stroke model

**Figure 5:**
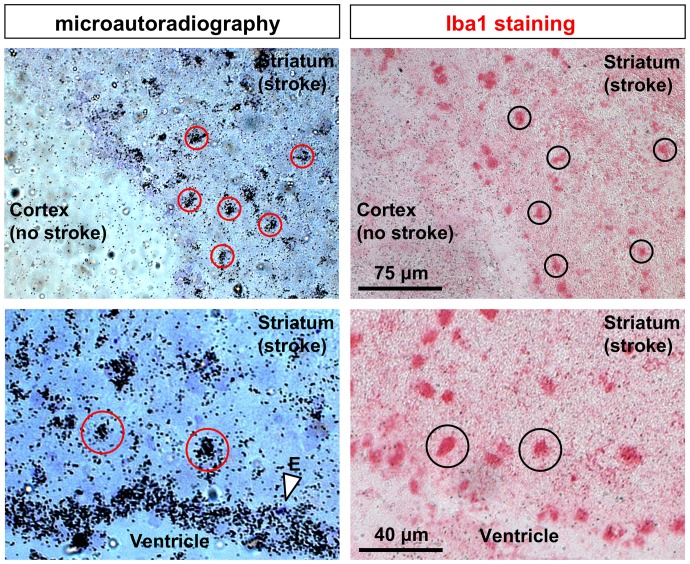
Microautoradiography and immunohistochemistry of [^3^H]DPA-714 72 h after tMCAO in mice. Binding of [^3^H]DPA-714 at the cellular level 72 h after stroke using microautoradiography (left column) and Iba1 immunohistochemistry (right column) is shown. Tissue sections were incubated with the tracer followed by Iba1 staining in adjacent sections. Tracer binding can be attributed to single cells positive for Iba1 staining (red circles in comparison to black circles). Strong tracer binding is also detected in the ependyma cells lining the ventricles (E, arrow).

To evaluate the distribution of [^3^H]DPA-714 on the cellular level, microautoradiography was performed by incubating tissue sections *ex-vivo* with the compound. Clusters of silver grains caused by [^3^H]DPA-714 binding were detected in the same areas as Iba1-positive cells (Fig. 5). Increased [^3^H]DPA-714 binding was also detected in the ependymal cells, reflecting the constitutively expressed TSPOs. Immunohistochemistry could only be performed on adjacent sections since Iba1 immunohistochemistry and microautoradiography cannot be conducted on the same slice.

## Discussion

The objective of this study was to specify the cellular binding of high-affinity TSPO ligand DPA-714 in a rat model of epilepsy and a mouse model of stroke. The results show that the ligand is a well-suited marker for specific detection of activated microglia/macrophages in these animal models.

DPA-714 was introduced as a TSPO ligand in 2008. James and colleagues showed that [^18^F]DPA-714 could cross the BBB and exhibited good uptake in the brain [19]. They also found an improved affinity of DPA-714 for TSPOs compared to PK11195. Using tissue extraction and gamma counter measurements in a rat model of Huntington's disease induced by striatal lesions with the exitotoxin quinolinic acid, they reported TBRs of about 8 between the ipsilateral and contralateral striatum. TBRs were reduced by pretreatment of the animals with the cold compounds DPA-714, DPA-713, or PK11195 [19]. In another study, Chauveau and coworkers employed [^18^F]DPA-714 in a striatal injury model using toxic concentrations of the synthetic glutamate receptor agonist α-amino-3-hydroxy-5-methyl-4-isoxazolepropionic acid (AMPA) [21]. In this model, tracer binding in the lesioned striatum was about 4-fold higher than that on the unaffected contralateral side. TBRs could be reduced to around 3 when applying [^11^C]DPA-713, or to around 2 when injecting [^11^C]PK11195 [21].

The results of our study confirm the high specificity of DPA-714 for detecting activated microglia/macrophages in two more, very different models of neuroinflammation. In the first part of our study, we employed a bilateral model of epilepsy leading to cell death and focal long-term activation of microglia. TBRs of around 2 were observed in the affected regions, the hippocampus and Entorhinal cortex, corresponding well with Ox42 immunostaining. Tracer uptake was effectively blocked by an excess of FEDAA1106 and DPA-714 (Fig. 1). In the second part of this study, acute unilateral striatal injury was induced by tMCAO. Increased tracer uptake into the affected ischemic striatum and damaged cortical regions were found, corresponding nicely with Iba1 staining (Fig. 2). The difference in the area of the lesion and the area of the [^3^H]DPA-714 signal, demonstrated in Fig. 2a (first and second row) could relate to the well-known penumbra, that is seen after stroke with microglia activation and/ or infiltrating macrophages. In Fig. 4a (second row), the same sections are given. The Iba1 staining of these sections shows nicely that there is no increase in the number of Iba1-positive cells in this area. However, these microglia are most likely activated and it is known that upon microglia activation the PBR gets upregulated [30]. This would in turn lead to increased binding in this area. The TBRs in this study were comparable to those observed in previous studies in rat models of stroke, which were around four [22], [23]. However, pre-injection of unlabeled DPA-714 or PK11195 in the stroke model in our study while decreasing the overall signal did not affect the TBRs. Tracer uptake decreased equally on both hemispheres which might be attributed to underdosing and/or timing of the pre-treatment.

Although BBB impairment is a hallmark of brain inflammation, it can influence lesional tracer uptake, and therefore, it may confound specific imaging of inflammation, since the imaging agent might have a higher lesional uptake due to unspecific extravasation. Thus, we investigated whether BBB impairment influences TBRs. To our knowledge, such experiments have not yet been performed in studies evaluating DPA-714. To this end, at the investigated time points in the stroke model, we demonstrate that neither the degree of BBB impairment (Fig. 3) nor the lesion volume (Fig. 2) correlate with tracer uptake (i.e. TBRs) in the brain. Therefore, passive tracer accumulation can be excluded as a major confounder in imaging brain inflammation with DPA-714 in the experimental stroke model used. It seems likely that this finding also applies to the use of inflammation imaging in other brain pathologies with BBB disturbance.

To further ascertain specificity of DPA-714 uptake, we used autoradiography combined with immunohistochemistry. In both Kainic acid-induced injury and tMCAO models, areas with increased tracer uptake correlated well with brain areas displaying Ox42- (Fig. 1) or Iba1-immunoreactivity (Fig. 2). In addition, in the tMCAO model, we showed increased tracer binding to cells in the lesioned area by high-resolution microautoradiography. The appearance and distribution of these cells resembled that of activated microglia/invading macrophages. The pattern of grain cluster distribution over the cells matched well with the pattern of Iba1-positive cells on the adjacent sections (Fig. 5). Additionally, it should be mentioned that astrocytes can express TSPOs as well [31]. However, Martin and colleagues previously described that TSPO expression on astrocytes after cerebral ischemia was comparatively low at 4 days after MCAO and is only important at later time points (up to 11 days after cerebral ischemia). The observed binding of the tracer to cells of the plexus choroideus, ependyma as well as to large vessels reflects their well-characterized increased expression of TSPOs [32]. This is also clearly demonstrated in our study and adds more evidence that the tracer binds specifically to TSPOs. Although signals from these brain regions might potentially reduce overall TBRs, this can be avoided by excluding the ventricular areas from the analysis.

A further confirmation of the high specificity of DPA-714 for imaging activated microglia/macrophages is given by the results of the time course after cerebral ischemia. Twenty-four hours after tMCAO, a lesion was clearly detectable, but neither a signal in the autoradiography nor in Iba1 staining could be found. At 96 h and 2 weeks after stroke, both Iba1-positive cells and tracer uptake increased in parallel (Fig. 4). These results are in accordance with the literature: In two studies from Martin and colleagues, PET imaging and autoradiography in a rat model of stroke demonstrated a significant increase in DPA-714 uptake in the ischemic lesion compared to the contralateral side at 7, 11, 15, and 21 days after ischemia, peaking at day 11 (TBRs of about 5) [22], [23]. Furthermore, Thiel and Heiss found an increase of TSPO expression in the CNS up to 4 weeks after human stroke [33]. From a pathophysiological standpoint, it is interesting to note that we found a delayed activation of microglia/macrophages (96 h) which lasted for a prolonged time period (2 weeks). We also found that microglial activation, BBB disturbance, and lesion size did not correlate in a straightforward manner. This concurs with studies using alternative approaches [34], [35] and disputes the notion of a simple model, which predicts that inflammation leads to BBB disruption and tissue damage. There appears to be a rather complex interaction of inflammation, BBB disruption, tissue damage, and potentially repair. Noninvasive imaging technologies such as PET or SPECT, e.g. using radiolabelled DPA-714, appear highly suited for unraveling these processes further facilitating stratification of patients to therapeutic interventions targeting inflammation.

In conclusion, the TSPO ligand DPA-714 seems to be a highly specific marker for both acute and chronic neuroinflammation. We showed that tracer uptake assessed by autoradiography and high-resolution micoautoradiography closely correlates with immunochemical staining. Remarkably, we are the first to show that the tissue in which uptake of [^3^H]DPA-714 occurs is identical to the Iba1-positive tissue. We also demonstrated that the [^3^H]DPA-714 signal does not correlate with BBB impairment or lesion volume. Passive ligand accumulation is therefore unlikely to confound imaging of microglia/macrophage activation in the brain.
